# The Multivariate Relationship Between Primary Anterior Cruciate Ligament Reconstruction Timing and Revision Rates: A 10-Year Analysis

**DOI:** 10.7759/cureus.21023

**Published:** 2022-01-07

**Authors:** Michael Brown, Gage A Hurlburt, Zachary A Koenig, David Richards

**Affiliations:** 1 Medicine, West Virginia School of Osteopathic Medicine, Lewisburg, USA; 2 Department of Medicine, West Virginia University School of Medicine, Martinsburg, USA; 3 School of Medicine, West Virginia University, Morgantown, USA; 4 Orthopedics, West Virginia University, Morgantown, USA

**Keywords:** timing, anterior crucial ligament injury, revision, reconstruction, anterior cruciate ligament

## Abstract

Background and objective

The optimal timing of anterior crucial ligament reconstruction (ACLR) remains a matter of controversy. A revision procedure is performed to improve knee function, correct instability, and enable a safe return to daily function when primary ACLR fails. The present study aimed to determine if the timing of primary ACLR is predictive of revision surgery.

Methods

All patients who underwent primary ACLR at the West Virginia University from January 2008 to December 2018 were identified. Patients were initially grouped into early (≤30 days) and late (>30 days) ACLR based on the onset of the initial injury. The major outcome measure of this study was the incidence of revision ACLR following primary ACLR.

Results

A total of 233 primary ACLRs were included. The incidence of ACLR revisions was 9.4%. The timing of primary ACLR, when categorized into early and late ACLRs, was not found to influence revision risk (p=0.384). Additionally, the damaged anatomical structures based on the postoperative diagnosis at the time of ACLR did not influence the odds of revision ACLR (p=0.9721).

Conclusion

Our study found that the timing of primary ACLR did not influence the revision rates when categorizing primary surgery time into early and late subgroups.

## Introduction

The anterior cruciate ligament (ACL) is a key structure providing stability to the knee, and its primary purpose is to limit the anterior translation of the tibia relative to the femur. Aside from this, its secondary role is to limit tibial rotation and varus or valgus stress, a principle known as the screw-home mechanism, which is essential for pivoting activities [1]. Injury to the ACL is one of the most common orthopedic injuries in the United States, affecting approximately 250,000 individuals annually with an annual incidence rate of 68.6 per 100,000 person-years for isolated ACL tears [2,3]. This injury is widely known to impact athletes and physically active individuals. The mechanism of injury is frequently associated with activities in which sudden stress is applied to the knee. Typically, it involves a non-contact injury where the tibia continues to translate anteriorly with the knee in a slightly flexed position, as seen in high-risk pivoting sports. Although ACL injuries are commonly encountered in orthopedic practice, it rarely occurs as an isolated disruption, and concomitant injury to other structures of the knee, such as the medial meniscus (MM) and lateral meniscus (LM), is common [4].

The goals of the management of ACL injuries are to restore knee function, prevent further knee injury, and optimize long-term quality of life [5]. Conservative treatment may be successful in certain populations; however, the intention of returning to a high-activity level may necessitate ACL reconstruction (ACLR) with the goal of regaining functional stability and maximum strength [6].

Nearly 8% of individuals who undergo primary ACLR end up requiring a subsequent revision procedure at some point in the future [7]. Causes for revision include, but are not limited to, nonanatomic tunnel placement, inadequate notchplasty, improper tensioning, insufficient graft material, arthrofibrosis, skeletal malalignment, varus/valgus instability, surgical techniques, and the type of graft utilized [8,9]. Compared to the primary reconstruction, revision surgeries are more complex as the existing femoral and tibial tunnels complicate the creation of new tunnels [10,11]. Furthermore, the recommended graft type varies with each procedure; moreover, surgeons have to choose between a single-stage or a two-stage revision [12]. 

Although reconstruction is the most common treatment for ACL injuries, the optimal timing of surgery remains uncertain and there continues to be ambiguity as to whether early ACLR is advantageous compared to delayed ACLR. Several studies have suggested that early ACLR is associated with an increased risk of arthrofibrosis [13-16]. Conversely, it is speculated that early reconstruction allows for earlier stabilization and restoration of knee biomechanics, minimizing the risk of concomitant knee pathology [17]. Moreover, concurrent injury to other anatomic structures of the knee at the time of the primary ACLR may necessitate revision surgery. If additional structures are left untreated, it may cause excessive stress on the graft, leading to graft failure [18,19]. The purpose of this study was to analyze the optimal timing of ACLR, specifically comparing revision rates in early (≤30 days) versus late (>30 days) ACLR from the injury onset. We hypothesize that early ACLRs will result in fewer revision rates compared to late ACLRs. Additionally, we aim to explore the impact of postoperative diagnosis and the type of injury pattern on primary and revision ACLR.

## Materials and methods

Ethical approval for this study was obtained from the West Virginia University Institutional Review Board (protocol number 2101226723). Patient data were extracted from the West Virginia University Medicine healthcare database. The inclusion criteria were as follows: male and female patients between 16 and 50 years of age who had undergone primary ACLR from January 1, 2008, to December 31, 2018, at various hospitals within the WVU Medicine system, with a minimum follow-up of two years. The exclusion criteria were as follows: a previous ACLR revision (because this study aimed to investigate if there is an association between primary surgery and the timing of injury onset), postoperative ACLR infections due to potential surgical complications, and workers’ compensation patients (due to lack of generalizability to the wider population). In addition to basic demographic information, several patient characteristics and risk factors for revision ACLR were investigated. This included the history of tobacco use, diabetes mellitus, hypertension, hyperlipidemia, participation in competitive sports, time from injury to primary surgery, and concomitant injuries. The patients were classified into two groups based on the time from injury to primary reconstructive surgery: early (≤30 days) or late (>30 days), based on the results of prior studies [3,6-8,14-15,17].

The major outcome measure of this study was the incidence of revision ACLR following primary ACLR. Patients who received revision ACLR at our institution during this timeframe were similarly identified via the electronic healthcare record system. In addition, the postoperative diagnosis was categorized based on the anatomical structures injured at the time of diagnosis [e.g., medial collateral ligament (MCL), MM, lateral collateral ligament (LCL), and LM].

The JMP software (version 16.1, SAS Institute Inc., Cary, NC) was used for the statistical analysis. Binomial logistic regression and Fisher’s exact test analyses were performed to assess the probability of revision rates concerning the timing of primary ACLR and concomitant knee injuries based on the postoperative diagnosis. This statistical test was also utilized to determine the association between postoperative diagnosis of concomitant injury and timing of primary ACLR. An additional analysis of the means for proportions was performed to explore the concomitant injury subgroups' proportion against the mean proportion for revision risk. The time from injury to surgery and concomitant injury represented the independent variables, with revision ACLR as the dependent variable. All measured variables were summarized with standard descriptive statistics such as frequency, median, and odds ratio. A p-value less than or equal to 0.05 was considered statistically significant.

## Results

**Figure 1:**
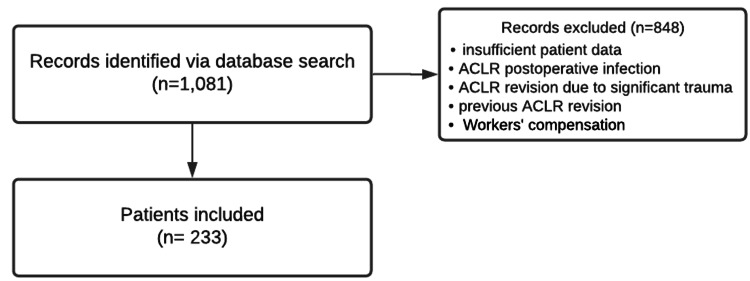
Flowchart detailing the selection criteria for our study ACLR: anterior crucial ligament reconstruction

A total of 1,081 patients who underwent primary ACLR were reviewed and 233 patients were ultimately included in this study (Figure 1). Many patients were not included in this study either due to their failure to meet the exclusion criteria or the absence of a significant amount of data in the electronic health records. The 233 primary ACLRs included 23 allografts, 45 bone-patellar tendon-bone autografts, and 165 hamstring autografts. Of the 233 patients included in the study, 22 patients underwent revision ACLR: two were treated initially with an allograft, one was treated initially with the bone-patellar tendon-bone autograft, and 19 were treated initially with the hamstring autograft.

Patient characteristics for the early ACLR (n=40) and late ACLR (n=193), as well as the revision ACLR (n=22) and non-revision ACLR (n=211) groups are summarized in Table 1. The frequency of revisions compared to non-revisions among the early and late primary ACLR groups was skewed, as the number of patients in the early (n=2) and late (n=20) primary ACLR subgroups who received a revision was small (Table 2). For those in the early group (≤30 days), the median number of days from injury to primary ACLR was 16.5, and it was 81 for the late group (>30 days). The median number of days from injury to primary ACLR for the revision ACLR group was 59.5, and it was 69 for the non-revision group.

**Table 1:** Patient characteristics and factors related to the early and late primary ACLRs and revision and no-revision ACLRs ACLR: anterior crucial ligament reconstruction

	Early ACLR (n=40)	Late ACLR (n=193)	X2 value	Revision ACLR (n=22)	Non-revision ACLR (n=209)	X2 value
Competitive athlete						
Yes	24	82	0.0431	17	89	0.056
No	16	111		5	120	
Tobacco use						
Yes	2	29	0.0608	1	30	0.0261
No	38	164		21	179	
Diabetes						
Yes	0	3	0.2860	0	3	0.4250
No	40	190		22	206	
Hypertension						
Yes	1	13	0.3654	1	13	0.7852
No	39	180		21	196	
Hyperlipidemia						
Yes	0	5	0.1674	0	5	0.5632
No	40	188		22	204	

When subgrouping patients based on early or late primary ACLR, Fisher’s exact test showed that the probability of the revision rates was not influenced by the timing of primary ACLR in relation to the injury (p=0.384) (Table 2). Thus, no significant correlation was found between the timing of primary ACLR and revision ACLR in terms of subgrouping patients into early or late ACLR. However, with respect to grouping the timing of ACLR into early and late groups, the data was skewed due to the disproportionate number of subjects in the late group compared to the early group.

**Table 2:** Total number of patients in the non-revision and revision ACLR groups who received early or late primary ACLR Fisher’s exact test analysis was performed to assess the probability of revision rates in relation to the timing of primary ACLR (p>0.05) ACLR: anterior crucial ligament reconstruction

	Non-revision ACLR	Revision ACLR
Early primary ACLR	38	2
Late primary ACLR	173	20
Fisher’s exact test two-sided probability ≤p		0.3844

**Figure 2:**
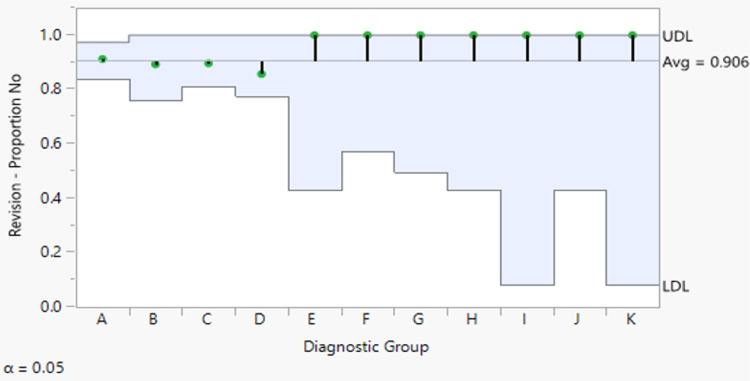
Analysis of the means for proportions of the postoperative diagnostic subgroups based on concomitant knee injury pattern for the revision ACLR group The proportions of revision ACLR for each of the diagnostic subgroups (i.e., categories for the injured structures at postoperative diagnosis) are plotted on the vertical axis. From top to bottom, the three horizontal lines represent the upper decision limit (UDL), the pooled proportion or overall average of the diagnostic subgroups, and the lower decision limit (LDL). No diagnostic group point (green circles) lies outside the UDL or LDL, and hence are not statistically significant from the overall proportion mean ACLR: anterior crucial ligament reconstruction

**Figure 3:**
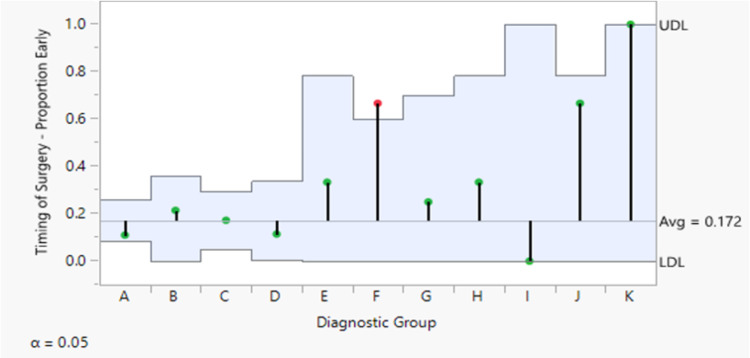
Total number of non-revision and revision ACLRs for the postoperative diagnostic subgroups based on concomitant knee injury patterns Fisher’s exact test analysis was performed on the probability of revision rates in relation to concomitant knee injuries based on the postoperative diagnosis (p>0.05) ACLR: anterior crucial ligament reconstruction

The anatomic structures involved as demonstrated in the postoperative diagnosis also did not influence the odds of revision ACLR (p=0.972). Each subgroup representative of concomitant knee injuries based on the postoperative diagnosis did not differ in proportion between non-revision ACLR and revision ACLR (Table 3). Additionally, the analysis of the means for proportions showed no true difference between any subgroup proportion and the overall proportion (Figure 2). Thus, the type of concomitant knee injury was not associated with a higher or lower risk for revision. However, Fisher’s exact test showed that the probability of the timing of primary ACLR was influenced by the concomitant knee injury pattern demonstrated in the postoperative diagnosis (p=0.005) (Table 4). The analysis of the means for proportions for subgroup F (ACL + MCL + LM injury) in particular exceeded the upper limit of the expected binomial distribution of the overall mean proportion (Figure 3).

**Table 3:** Total number of early and late ACLR cases for the postoperative diagnostic subgroups based on concomitant knee injury patterns Fisher’s exact test was performed on the association between postoperative diagnosis of concomitant injury and timing of primary ACLR (p<0.05) ACLR: anterior crucial ligament reconstruction; LCL: lateral collateral ligament; LM: lateral meniscus; MCL: medial collateral ligament; MM: medial meniscus; PCL: posterior cruciate ligament

		Structure(s) injured	Early ACLR	Late ACLR
Postoperative diagnostic subgroup	A	ACL	10	81
	B	ACL + MM	6	22
	C	ACL + LM	10	48
	D	ACL + MM + LM	4	31
	E	Multiligamentous	1	2
	F	ACL + MCL + LM	4	2
	G	ACL + MCL	1	3
	H	ACL + MCL + MM	1	2
	I	ACL + MCL + PCL + LM	0	1
	J	ACL + LCL	2	1
	K	ACL + LCL + PCL	1	0
Fisher’s exact test two-sided probability ≤p			0.0050	

**Table 4:** Total number of non-revision and revision ACLRs for the postoperative diagnostic subgroups based on concomitant knee injury patterns Fisher’s exact test analysis was performed on the probability of revision rates in relation to concomitant knee injuries based on the postoperative diagnosis (p>0.05) ACLR: anterior crucial ligament reconstruction; LCL: lateral collateral ligament; LM: lateral meniscus; MCL: medial collateral ligament; MM: medial meniscus; PCL: posterior cruciate ligament

		Structure(s) injured	Non-revision ACLR	Revision ACLR
Postoperative diagnostic subgroup	A	ACL	83	8
	B	ACL + MM	25	3
	C	ACL + LM	52	6
	D	ACL + MM + LM	30	5
	E	Multiligamentous	3	0
	F	ACL + MCL + LM	6	0
	G	ACL + MCL	4	0
	H	ACL + MCL + MM	3	0
	I	ACL + MCL + PCL + LM	1	0
	J	ACL + LCL	3	0
	K	ACL + LCL + PCL	1	0
Fisher’s exact test two-sided probability ≤p			0.9721	

## Discussion

We sought to determine whether reconstructing the ACL in an acutely injured knee irrespective of its preoperative status would lead to less revision rates in the future. The assumption was that a shortened length of time between the injury date and surgical repair prevented further injury and reduced the risk of a future traumatic event, thereby decreasing the risk of additional inflammatory response and damage [20]. The present study demonstrated that the rate of revision is not influenced by the timing of the ACLR.

There is limited supporting literature regarding the association between the timing of initial ACLR and revision ACLR. Nevertheless, Andernord et al.’s study determined that the timing of ACLR was not an independent predictor of revision [21]. Conversely, Snaebjörnsson et al. discovered that there is a higher risk of revision if reconstruction took place within three months of the ACL injury and six months of ACL injury in the hamstring tendon autograft and patellar tendon autograft, respectively [22]. Unlike our hypothesis, Snaebjörnsson et al. proposed that patients in the delayed group have more time to adapt to their injured knee and reduce activities that may lead to subsequent ACLR revision. Similarly, in a study performed by Cristiani et al., ACLR less than 12 months from the time of injury had an increased odds of revision ACLR within two years of primary ACLR [23]. While there is still a lack of consensus about the optimal timing of ACLR, our study suggests that revision rates were not influenced by the timing of ACLR.

Other studies have shown a link between the timing of ACLR and the development of concomitant knee injuries. It is important to consider knee structures other than the ACL that may play a role in determining the timing of surgery. Another topic of debate in the literature regarding ACLR is whether early or late reconstruction will optimize patient outcomes in terms of restoring knee function and minimizing injury to other anatomic structures. Delaying surgical reconstruction can potentially lead to subsequent episodes of instability and may result in concomitant knee pathologies. Late reconstruction may increase the risk of developing meniscal and cartilage injuries [24]. In a study performed by Demirağ et al., an increased time interval between injury to surgery increased the incidence of meniscal and osteochondral lesions [25]. Of note, the meniscal and osteochondral lesions occurred not at the time of injury, but during the period between original injury and surgical reconstruction as evidenced by early post-traumatic MRI or diagnostic arthroscopy conducted at the time of injury. This is supported by several studies indicating an increased incidence of meniscal tears in individuals undergoing ACLR more than 12 months from injury [26,27]. A study performed by Kennedy et al. showed that patients who received reconstruction six months after the injury date were noted to have an increased risk of developing degenerative change [27]. This is clinically important as the initial ACL injury can subsequently cause meniscal injury, which could alter the mechanics of the knee and accelerate the degenerative process. Similarly, Granan et al. concluded that the risk of a cartilage lesion in the adult knee increased as more time elapsed from injury date to surgery [28]. Early ACLR may be advantageous to preserve the meniscus and reduce the risk of osteoarthritis [24].

We found a connection between the timing of primary ACLR and the postoperative diagnosis. The contingency analysis (Table 4) demonstrated that postoperative diagnosis did influence the timing of ACLR (p=0.005). In fact, the likelihood of early primary surgery was more likely in a certain subset of the population. Specifically, subgroup F; individuals who suffered a simultaneous ACL, MCL, and LM injury were more likely to receive early primary ACL reconstruction. Although we cannot extrapolate the reason as to why this occurred, it is worth mentioning as it could aid potential research in the future. It is possible that these involved structures at the time of injury could result in greater instability or decline in daily function and thus warranted the decision to operate earlier.

There were no significant correlations between the postoperative diagnosis at the time of primary ACLR and the risk of revision. As we subcategorized each postoperative diagnosis on the type of injury pattern (Figure 2), none of them significantly affected the risk of revision. This suggests that concomitant knee injuries at the time of the initial ACLR were not associated with the need for future revision. Furthermore, revision rates were not influenced by a concomitant knee injury that excluded ACL involvement alone.

There is still a level of ambiguity as to whether subgrouping patients into early or late primary ACLR is advantageous, as the correlation between surgical timing for primary ACLR and indication for revision ACLR was not supported in our study. Ultimately, we suggest that the decision to undergo reconstruction is a matter of clinical judgment and the goals of the patient.

Several limitations in our study should be acknowledged. Firstly, the sample size was small (n=233), especially for subjects who received early primary ACLR (n=40), as well as the total number of patients requiring revision ACLR (n=22). This most likely impacted the study’s power and precluded the ability to obtain more significant results for the primary and secondary objectives of the study. Future studies should try to include a cohort large enough (i.e., more subjects with revision surgery) to better analyze the results. This study represents one of the few studies utilizing retrospective data concerning this topic. It is our understanding that the previous literature regarding the timing of primary ACLR and the potential impact it has on future revision was scarce. Additionally, other independent factors could have influenced the outcome of our results. For instance, most patients (70.8%) included in the study received hamstring autograft and it was difficult to account for surgical error, both of which could have impacted the surgical outcome and revision rate. For this reason, we recommend that future studies match subjects who had revision ACLR based on graft utilized, structures injured, and timing of primary ACLR. We also propose for further investigations or randomized control trials to include a standardized rehabilitation protocol for all patients who received ACLR regardless of the timing of primary reconstruction. This would likely minimize an additional potential influence on revision rates. Lastly, it is possible that the timeframe of the study did not allow for adequate capture of revision surgery as the last set of patients were recruited in 2018.

## Conclusions

We found no difference in the revision rates by grouping patients into early and late primary ACLR. There may not be a univariate answer for the question related to the optimal timing for primary ACLR, and other factors should also be accounted for when ACLR is considered, such as patient goals, additional anatomical structures involved, surgical technique, graft choice and fixation, and rehabilitation programs.
